# Distinguishing Different Stages of Parkinson’s Disease Using Composite Index of Speed and Pen-Pressure of Sketching a Spiral

**DOI:** 10.3389/fneur.2017.00435

**Published:** 2017-09-06

**Authors:** Poonam Zham, Dinesh K. Kumar, Peter Dabnichki, Sridhar Poosapadi Arjunan, Sanjay Raghav

**Affiliations:** ^1^School of Engineering, RMIT University, Melbourne, VIC, Australia; ^2^Dandenong Neurology, Melbourne, VIC, Australia

**Keywords:** Parkinson’s, kinematic feature, speed, pen-pressure, dynamic handwriting features

## Abstract

The speed and pen-pressure while sketching a spiral are lower among Parkinson’s disease (PD) patients with higher severity of the disease. However, the correlation between these features and the severity level (SL) of PD has been reported to be 0.4. There is a need for identifying parameters with a stronger correlation for considering this for accurate diagnosis of the disease. This study has proposed the use of the Composite Index of Speed and Pen-pressure (CISP) of sketching as a feature for analyzing the severity of PD. A total of 28 control group (CG) and 27 PD patients (total 55 participants) were recruited and assessed for Unified Parkinson’s Disease Rating Scale (UPDRS). They drew guided Archimedean spiral on an A3 sheet. Speed, pen-pressure, and CISP were computed and analyzed to obtain their correlation with severity of the disease. The correlation of speed, pen-pressure, and CISP with the severity of PD was −0.415, −0.584, and −0.641, respectively. Mann–Whitney *U* test confirmed that CISP was suitable to distinguish between PD and CG, while non-parametric *k*-sample Kruskal–Wallis test confirmed that it was significantly different for PD SL-1 and PD SL-3. This shows that CISP during spiral sketching may be used to differentiate between CG and PD and between PD SL-1 and PD SL-3 but not SL-2.

## Introduction

Parkinson disease is associated with movement disorder symptoms, such as tremor, rigidity, bradykinesia, and postural instability (1). The manifestation of bradykinesia and rigidity is often in the early stages of the disease (2). These have a noticeable effect on the handwriting and sketching abilities of patients, and micrographia has been used for early-stage diagnosis of Parkinson’s disease (PD) (3–6). While handwriting of a person is influenced by a number of factors such as language proficiency and education, sketching of a shape such as the spiral has been found to be non-invasive and independent measure (7).

The association of handwriting and sketching of the spiral has been established in PD in early stages (8, 9). However, one shortcoming in the use of handwriting or sketching is the need for an expert to interpret the sketches, especially in the early stages of the disease. With the availability of digital devices that are suitable for recording hand-sketching, there is the potential for machine based assessment of writing and sketching. These devices are also suitable for obtaining the dynamic features of handwriting, which are suitable for real-time and reliable analysis (10, 11). These features can be obtained automatically allowing rapid on-line assessment of patients (12) and developed for applications such as biometrics (13) and indicative markers for PD (14).

The kinematics of spiral drawing indicates physiological parameters such as the amplitude of tremor (15) and extent of bradykinesia (16) and dyskinesia (17). It has been shown to successfully differentiated between distal and proximal tremors (18) and between control group (CG) and PD (9, 19).

Identifying the level of severity is important for optimal clinical decision. Saunders et al. successfully quantified drawing of the spiral and identified the speed to be associated with the severity levels (SLs) of the disease among PD patients (7). While this is extremely useful to demonstrate the association, the maximum correlation coefficient reported was only 0.4.

The pen-pressure of PD patients is another feature that is associated with sketching and has been found to reduce compared with CG (14). While this is suitable for distinguishing between healthy subjects and people with the significant severity of the disease, it has not been shown to be suitable for distinguishing between different stages of the disease.

The aim of this work was to establish a reliable computer-based spiral sketching method for assessment of the severity of the disease. This study has investigated the dynamics of sketching a spiral to distinguish between healthy subjects and PD patients with different levels of severity and proposes a new feature with stronger association with the severity of the disease. The earlier studies have established that speed and pen-pressure during sketching reduce with the advancement of the disease (4, 14) but did not consider the combination of these two parameters. This study used the scalar product of these two features to obtain the Composite Index of Speed and Pen-pressure (CISP) of sketching and tested this against the severity of the disease.

## Materials and Methods

The differences in the dynamics of sketching a guided spiral were investigated using a CG and PD patients based on Unified Parkinson’s Disease Rating Scale (UPDRS) and modified H and Y rating scale severity. Proprietary software was developed that automatically recorded the pen-pressure and position of the pen and measured the average speed, pen-pressure, and CISP of sketching. Statistical and correlation analyses were performed using the SPSS software.

### Subjects

Fifty-five age-matched volunteers ranging from UPDRS = 0 (CG) to severely affected patients (UPDRS >24) were studied. All PD patients were recruited from PD outpatient clinic at Dandenong Neurology, Melbourne, Australia, while the CG subjects were from multiple aged-care facilities using word-of-mouth and appropriately located posters. All subjects were right hand dominant. The CG subjects were recruited to approximately match the age distribution and gender of the PD patients. The exclusion criteria were clinically observed or self-reported skeletal injuries and neurological and muscular-skeletal diseases, excess Levodopa medication that caused dyskinesia. For the PD patients who were on levodopa treatment, the experiments were conducted while they were in the “on” stage. The demographic and clinical data are shown in Table 1.

**Table 1:** Demographics and clinical information of participants.

	Control group	Parkinson’s disease
**Demographics**
Number of Subjects, *n*	28	27
Age, years	71.32 ± 7.21	71.41 ± 9.37
Gender male/female	21/6	22/6
**Clinical information**
Disease duration, years	–	6.7 ± 4.44
Unified Parkinson’s Disease Rating Scale-III	–	17.59 ± 7.69

*Values are represented as mean ± SD*.

The severity of motor symptoms for all participants was assessed by a qualified neurologist using part III of UPDRS Scale (Q18-31), and overall PD stage assessment was done using Modified Hoehn and Yahr (H&Y) Scale. SL 0 indicates the CG with no PD symptoms. Based on UPDRS III (20) and modified H and Y rating scale (21, 22) further groups were labeled as SL: 1–3 (see Table 2). None of the patients were in the late stage of the disease or were bedridden.

**Table 2:** Groups based on severity levels (SLs).

SL	Number of subjects	Unified Parkinson’s Disease Rating Scale (UPDRS) Sec III score (0–56)	UPDRS Mean ± SD	Modified H&Y stages (Sec V)
0	28	0	–	0
1	12	>0 and <15	10.75 ± 2.18	1, 1.5
2	8	≥15 and ≤23	18.38 ± 2.83	2, 2.5
3	7	> 24	28.43 ± 2.64	≥3

### Data Recording

The experimental protocol was approved by RMIT University Human Research Ethics Committee and in accordance with Declaration of Helsinki (revised 2004). All participants were informed about the experiment, and they provided their oral and written consent before the start of the experiment.

One shortcoming in the use of spiral drawing is the significant variation between the different studies. One option is where the participants are free to draw the spiral, which has the disadvantage that there is significant inter-participant variability (23, 24). The other template is by providing a continuous spiral for the participants to trace, which however is not feasible for many elderly participants. Another option is the use of light guided spiral (25), which was however found to be unsuitable for the elderly patients.

**Figure 1:**
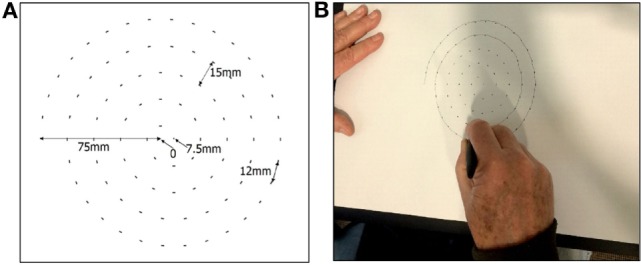
**(A)** Dot-guided spiral and **(B)** participant drawing the spiral.

This study overcame the abovementioned shortcomings and the participants used bright dots to guide the participants to sketch the spiral at their own speed and without emphasis on its accuracy (Figure 1). This is comfortable for all the participants, overcomes the potential bias due to shape based visual feedback, and ensures consistency of the number of circles made by all participants.

The sketching of the spiral was recorded using commercially available A3 size tablet (Wacom Intuos Pro Large). The A3 paper was placed on the tablet, and Ink pen (Wacom Intuos ink pen) was used to sketch the spiral using guiding dots (Figure 1). This pen senses the location of contact, *x* and *y*, and the pressure, *pr*, between the tip and the paper.

The center of the sheet was considered to be the (0,0) point. The guided Archimedean spiral was made using Adobe Illustrator software. It had 4.5 revolutions and a maximum radius 75 mm with an incremental increase in the diameter of 15 mm. The spiral was sketched in the clock-wise direction, the starting point was −75,0 mm, the end point was 7.5,0 mm, and the distance between two consecutive dots in the spiral was 12 mm (Figure 1A). The dots were of 2 mm each so that it was clearly visible. Participants were allowed to sketch the spiral at their own speed starting from the outside (−75 mm, 0) till starting point (Figure 1B) (10).

Proprietary software was developed and used to record and analyze the data in real time. The recorded data contain the following information:

- Location (*x*, *y*) with *x_n_* and *y_n_* corresponding to the *n*th sample.
  *x*, *y* are received in millimeter.
- pen-pressure (*pr*) recorded by the pen.
  *pr* is unit-less with the range; 0–1,024 units and
- *n*, the sample number.
  The sampling rate was 133 Hz.

### Computation of Features

The data were segmented to identify segments between each pen-down and corresponding pen-up; pen-down identified based on *pr* > 0, and given an index label, *i* with *m_i_* being the total number of samples of the segment.

The total length of each segment, *d_i_*, was computed (Eq. 1) and segments that corresponded to less than 0.5 mm of distance traveled were considered as noise and deleted. The remaining *N* segments and parameters were relabeled, *i* (1 to *N*).

The total time duration for each segment is *T_i_* (Eq. 2). Average speed (millimeter/second) was obtained using Eq. 3. The speed for each segment was weighted with the length of that segment to get the weighted average speed, ${\bar{S}}_{w}$, and was computed (Eq. 4).

Mean pen-pressure $\left({\bar{\mathrm{pr}}}_{i}\right)$ was obtained for each segment and the corresponding weighted average was calculated using Eq. 5. Average CISP of the spiral sketching, ${\bar{I}}_{\mathrm{spr}}$ was calculated using Eq. 6. This is the product of ${\bar{S}}_{w}$ and weighted pen-pressure, ${\bar{\mathrm{pr}}}_{w}$.

(1) $$d_{i}=\sum_{n=0}^{m_{i}}\sqrt{{\left(x_{n+1}-x_{n}\right)}^{2}+{\left(y_{n+1}-y_{n}\right)}^{2}}$$

(2) $$T_{i}=\frac{m_{i}}{133}$$

(3) $$s_{i}=\frac{d_{i}}{T_{i}}$$

(4) $${\bar{S}}_{w}=\frac{\sum_{i=1}^{N}d_{i}s_{i}}{\sum_{i=1}^{N}d_{i}}$$

(5) $${\bar{\mathrm{pr}}}_{w}=\frac{\sum_{i=1}^{N}d_{i}\bar{pr_{i}}}{\sum_{i=1}^{N}d_{i}}$$

(6) $${\bar{I}}_{\mathrm{spr}}={\bar{S}}_{w}*{\bar{\mathrm{pr}}}_{w}$$

### Statistical Methods

Statistical analysis was performed using the non-parametric tests to determine the statistical significance of the difference between the groups (22, 26). Mann–Whitney test was conducted to determine the difference in the parameter value between PD and CG. Two tests were conducted.

- Mann–Whitney *U* test is suitable for two group analyses and was conducted to determine the difference in the parameter values between PD and CG.
- Non-parametric *k*-sample Kruskal–Wallis test was performed to distinguish between different SLs among the PD as it contains three groups.

The sensitivity, specificity, and accuracy of classification were computed, and these were used to generate the receiver operating characteristics (ROC). The area under the ROC curve (AUC) was computed to determine the ability of the technique to differentiate between PD and CG.

Spearman rank-order correlation coefficient analysis was conducted to determine the association between the groups based on SL for the three features corresponding to the dynamics of sketching the spiral.

## Results

**Figure 2:**
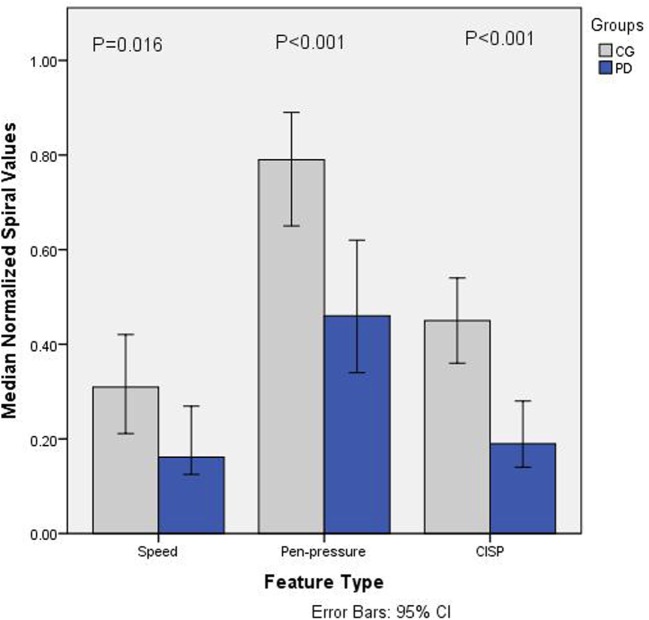
Barchart showing median normalized values (0–1) of speed, pen-pressure, and Composite Index of Speed and Pen-pressure (CISP) for Parkinson’s disease (PD) and control group (CG).

Figure 2 shows the normalized value (0–1) of weighted average speed $\left({\;\bar{\;S}}_{w}\right)$, the average of pen-pressure $\left({\bar{\mathrm{pr}}}_{w}\right)$ and average CISP of sketching $\left({\bar{I}}_{\mathrm{spr}}\right)$ for PD and CGs. It is observed that for all the features, the values for PD are lower compared to CG. Statistical analysis results using Mann–Whitney *U* test shows statistical significance difference for each value; speed *U* = 233, *p* = 0.0159; pen-pressure *U* = 139, *p* < 0.001, and CISP *U* = 130, *p* < 0.001. These indicate that there is significant group difference between PD and CG.

The specificity and sensitivity were calculated after the classification and area under ROC curve. The results showed the classification accuracy of 79.1% with area under ROC curve as 86.2% for CISP, whereas the combined features of speed and pen-pressure showed the classification accuracy of 68.2% with area under ROC curve as 83.2%. This shows that while there is significant group difference for all the features, the classification accuracy for CISP is much higher compared to the other two features.

**Figure 3:**
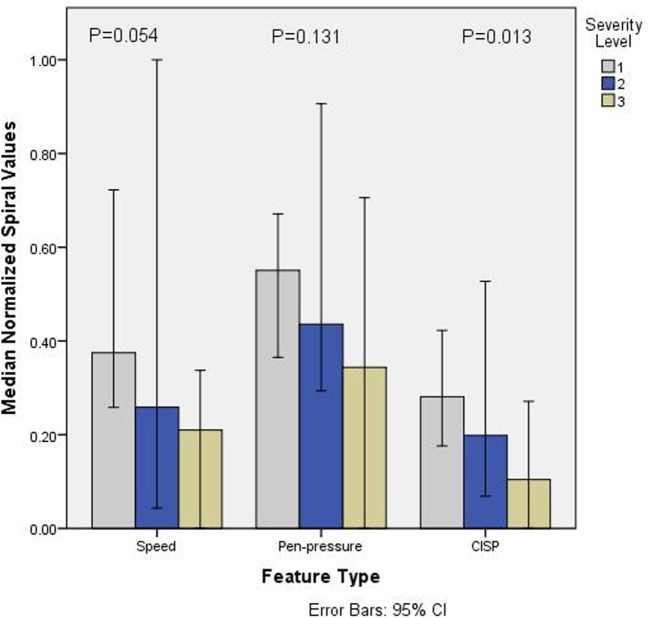
Barchart showing median normalized values (0–1) of speed, pen-pressure, and Composite Index of Speed and Pen-pressure (CISP) versus severity level (SL) (1–3) of Parkinson’s disease.

Figure 3 shows the normalized values (0–1) for all the three features for different groups of PD based on SL. It is observed that the values of all the parameters reduced with SLs. Non-parametric *k*-sample Kruskal–Wallis test shows that there is statistically significant difference between groups for CISP (χ^2^(3) = 8.753, *p* = 0.013), whereas speed (χ^2^(3) = 5.907, *p* = 0.052) and Pen-Pressure (χ^2^(3) = 4.064, *p* = 0.131) did not show statistically significant difference (α = 0.05). This indicates that speed and pen-pressure by themselves are not suitable for differentiating between the different levels of severity. Follow-up test was conducted to evaluate pairwise differences among the three PD groups, controlling for Type I error across tests by using the Bonferroni approach. A pairwise comparison showed a significant difference (*p* = 0.009) for SL-3 and SL-1. However, no significant difference was found for SL-2/SL-1 (*p* = 0.283) and SL-1/SL-3 (*p* = 0.709).

Spearman rank-order correlation coefficient analysis between all groups and the three parameters corresponding to the dynamics of sketching the spiral; ${\bar{S}}_{w}$, ${\bar{\mathrm{pr}}}_{w}$, and ${\bar{I}}_{\mathrm{spr}}$ have been tabulated in Table 3 (A) while for PD patients only are shown in Table 3 (B). From Table 3 (A), it is observed that *r_s_* = −0.421 for speed and *r_s_* = −0.584 for pen-pressure while for CISP *r_s_* = −0.641.

**Table 3:** Spearman correlation coefficients of spiral for dynamic features.

Spiral	Speed	Pen-Pressure	Composite Index of Speed and Pen-pressure
**(A) Severity level (SL) 0–3 considered**			
SL (group)	−0.421**(0.001)	−0.584**( <*0.001)*	−0.641**( <*0.001)*
Unified Parkinson’s Disease Rating Scale (UPDRS) Sec III	−0.415**(0.002)	−0.591**( <*0.001)*	−0.650** *(* <*0.001)*
H&Y Scale (V)	−0.405** *(0.002)*	−0.580**( <*0.001)*	−0.631** *(* <*0.001)*
S&E scale (VI)	0.455*(0.017)	0.466*(0.014)	0.631**( <*0.001)*
**(B) Only Parkinson’s disease considered (SL 1–3)**			
SL (group)	−0.475* *(0.012)*	−0.383* *(0.49)*	−0.568** *(0.002)*
UPDRS Sec III	−0.412* *(0.033)*	−0.404* *(0.037)*	−0.573** *(0.002)*
H&Y scale (V)	−0.394* *(0.042)*	−0.356 *(0.068)*	−0.518** *(0.006)*
S&E scale (VI)	0.455* *(0.017)*	0.466* *(0.014)*	0.631** *(* <*0.001)*

r_s_ (*p*-values) values of Spearman correlation coefficients where *n* = 55 (A) and *n* = 27 (B), correlation is significant at the 0.01 *level* (2-*tailed*)** and 0.05 *level* (2-*tailed*)*, −ve values indicate correlation.

The coefficient, *r_s_*, for CISP = −0.568, whereas speed and pen-pressure show *r_s_* = −0.475 and *r_s_* = −0.383, respectively, when the three PD groups and without CG were considered. This indicates that the discrimination between the three levels of severity of PD by CISP and speed was moderate (range 0.4–0.59), while it was weak (0.2–0.39) for pen-pressure. This was found to be valid for grouping based on UPDRS (Section III), Modified H&Y (Section V) or Schwab & England (S&E) scale.

## Discussion

Bradykinesia in PD patients causing reduced speed is well recognized (27). Mann–Whitney *U* test has shown that our findings are in line with earlier finding who found that PD patients sketched the spiral slower than the healthy subjects. Our findings are also in line with earlier finding, which shows kinematic feature; speed, and pen-pressure are reduced in PD compared with CG (14, 28).

Spearman rank-order correlation coefficients of the association of speed and pen-pressure with the severity of the disease are in line with Saunders et al. and show a correlation of 0.4 (7). The results show that speed and pen-pressure were lower with patients having higher severity of the disease (Figure 3). This extends the earlier findings (4, 14) who had reported similar difference and attributed to the complexity of task (29) and bradykinesia (27). However, this has not been uniformly accepted (30). This study has shown that while there is a reduction in the mean values of speed and pen-pressure with the severity of the disease, this is not statistically significant. This shows that speed or pen-pressure is not suitable to differentiate between the severities of PD. Thus, while these are suitable for differentiating between PD and CG, these are not suitable to differentiate based on the severity of PD.

This study has shown that the composite feature proposed in this study is suitable for differentiating between PD and CG and between SL-1 and SL-3. CISP is the product of the two features, speed of sketching and pen-pressure during sketching. The results show that when considering two group problems, PD and CG, the classification accuracy, sensitivity, specificity, and area under ROC curve is higher for CISP than for the individual features, speed and pen-pressure. It is also seen that Spearman rank ordered correlation coefficient for CISP is stronger compared to speed and pen-pressure when comparing between patients with different levels of severity of PD (Table 3).

The advantage of the proposed method is that it is suitable for being used without supervision. The features (Eqs 1–6) are independent of the starting point of the spiral (23) and the computation of the features is in real time. The test requires simple instructions and is not dependent on the language skills of the patient, and the complete test takes around l0 minutes.

## Conclusion

This study has shown that speed, pen-pressure, and CISP of sketching of a spiral are negatively correlated with the severity of PD. While these three features were significantly affected by the severity of the disease, the correlation was strongest with the CISP of sketching. Statistical analysis showed a significant difference of CISP between SLs 1 and 3 but not for speed and pen-pressure. However, CISP was not able to differentiate between SL1 and SL2 or between SL2 and SL-3.

This study proposes that average CISP of drawing a spiral for monitoring PD patients and assessing the severity of their disease. However, this study suffers from two limitations, which are the basis for the next studies, increase in patient numbers and longitudinal study. One limitation in this study is that number of patients for individual SLs was relatively small, 7, 8, and 12. Based on CISP calculations, there is a need to extend it so that the sample size for each SL is greater than 20. The second limitation of this study is that it is a cross-sectional study. To confirm the suitability of this method for monitoring the patients, a longitudinal study that will monitor the patients over the progression of their disease needs to be conducted.

## Ethics Statement

The experimental protocol was approved by RMIT University Human Research Ethics Committee and in accordance with Declaration of Helsinki (revised 2004). All participants were informed about the experiment, and they provided their oral and written consents before the start of the experiment.

## Author Contributions

PZ involved in data acquisition, data analysis, software design and development for collecting and analysis of data, statistical analysis, and drafting the article. DK involved in concept of work, drafting the article, critical revision of an article, and final approval of an article. PD contributed to concept of work, designing, selection of analytical tools, and critical revision of an article. SA contributed to statistical analysis, selection of analytical tool, and drafting and revision of an article. SR is a senior neurologist who helped in clinical assessment, data collection, design work, and critical revision of an article.

## Conflict of Interest Statement

The authors declare that the research was conducted in the absence of any commercial or financial relationships that could be construed as a potential conflict of interest.

## References

[1] Jankovic J Parkinson’s disease: clinical features and diagnosis. J Neurol Neurosurg Psychiatry (2008) 79(4):368–76. 10.1136/jnnp.2007.131045 18344392

[2] Politis M Wu K Molloy S G Bain P Chaudhuri K Piccini P. Parkinson’s disease symptoms: the patient’s perspective. Mov Disord (2010) 25(11):1646–51. 10.1002/mds.23135 20629164

[3] Poluha P Teulings H-L Brookshire R Handwriting and speech changes across the levodopa cycle in Parkinson’s disease. Acta Psychol (1998) 100(1):71–84. 10.1016/S0001-6918(98)00026-2 9844557

[4] Rosenblum S Samuel M Zlotnik S Erikh I Schlesinger I Handwriting as an objective tool for Parkinson’s disease diagnosis. J Neurol (2013) 260(9):2357–61. 10.1007/s00415-013-6996-x 23771509

[5] Letanneux A Danna J Velay JL Viallet F Pinto S. From micrographia to Parkinson’s disease dysgraphia. Mov Disord (2014) 29(12):1467–75. 10.1002/mds.25990 25156696

[6] Smits EJ Tolonen AJ Cluitmans L van Gils M Conway BA Zietsma RC Standardized handwriting to assess bradykinesia, micrographia and tremor in Parkinson’s disease. PLoS One (2014) 9(5):e97614. 10.1371/journal.pone.0097614 24854199 PMC4031150

[7] Saunders-Pullman R Derby C Stanley K Floyd A Bressman S Lipton RB Validity of spiral analysis in early Parkinson’s disease. Mov Disord (2008) 23(4):531–7. 10.1002/mds.21874 18074362

[8] Graça R e Castro RS Cevada J ParkDetect: Early Diagnosing Parkinson’s Disease. Lisboa: IEEE (2014). p. 1–6.

[9] San Luciano M Wang C Ortega RA Yu Q Boschung S Soto-Valencia J Digitized spiral drawing: a possible biomarker for early Parkinson’s disease. PLoS One (2016) 11(10):e0162799 10.1371/journal.pone.0162799 27732597 PMC5061372

[10] Zham P Kumar DK Dabnichki P Raghav S Keloth SM Dynamic handwriting analysis for assessing movement disorder. In: 13th International Conference of Applied Computing Manheim, Germany: IADIS (2016).

[11] Sisti JA Christophe B Seville AR Garton AL Gupta VP Bandin AJ Computerized spiral analysis using the iPad. J Neurosci Methods (2017) 275:50–4. 27840146 10.1016/j.jneumeth.2016.11.004 PMC5308231

[12] Surangsrirat D Thanawattano C Android application for spiral analysis in Parkinson’s disease. 2012 Proceedings of IEEE Southeastcon Orlando, FL: IEEE (2012). p. 1–6.

[13] Unnikrishnan P Kumar DK Arjunan SP Class specific dynamic feature selection technique—towards human movement based biometrics application. 2013 ISSNIP Biosignals and Biorobotics Conference (BRC) Rio de Janerio: IEEE (2013). p. 1–4.

[14] Drotár P Mekyska J Rektorová I Masarová L Smékal Z Faundez-Zanuy M. Evaluation of handwriting kinematics and pressure for differential diagnosis of Parkinson’s disease. Artif Intell Med (2016) 67(1):39–46. 10.1016/j.artmed.2016.01.004 26874552

[15] Kraus PH Hoffmann A. Spiralometry: computerized assessment of tremor amplitude on the basis of spiral drawing. Mov Disord (2010) 25(13):2164–70. 10.1002/mds.23193 20572156

[16] Banaszkiewicz K Rudzińska M Bukowczan S Izworski A Szczudlik A Spiral drawing time as a measure of bradykinesia. Neurol Neurochir Pol (2008) 43(1):16–21. 19353440

[17] Liu X Carroll CB Wang S-Y Zajicek J Bain PG. Quantifying drug-induced dyskinesias in the arms using digitised spiral-drawing tasks. J Neurosci Methods (2005) 144(1):47–52. 10.1016/j.jneumeth.2004.10.005 15848238

[18] Wang S Bain PG Aziz TZ Liu X. The direction of oscillation in spiral drawings can be used to differentiate distal and proximal arm tremor. Neurosci Lett (2005) 384(1):188–92. 10.1016/j.neulet.2005.04.084 15896904

[19] Stanley K Hagenah J Brüggemann N Reetz K Severt L Klein C Digitized spiral analysis is a promising early motor marker for Parkinson disease. Parkinsonism Relat Disord (2010) 16(3):233 10.1016/j.parkreldis.2009.12.007 20079674 PMC2862351

[20] Fahn S Elton RL Unified rating scale for Parkinson’s disease. In: Fahn S Marsden CD, editors. Recent Developments in Parkinson’s Disease. Florham Park, New York: Macmillan (1987). p. 153–63.

[21] Hoehn MM Yahr MD Parkinsonism: onset, progression, and mortality. Neurology (1998) 50(2):318–318. 10.1212/WNL.50.2.318 9484345

[22] Goetz CG Poewe W Rascol O Sampaio C Stebbins GT Counsell C Movement Disorder Society Task Force report on the Hoehn and Yahr staging scale: status and recommendations the Movement Disorder Society Task Force on rating scales for Parkinson’s disease. Mov Disord (2004) 19(9):1020–8. 10.1002/mds.20213 15372591

[23] Wang H Yu Q Kurtis MM Floyd AG Smith WA Pullman SL Spiral analysis—improved clinical utility with center detection. J Neurosci Methods (2008) 171(2):264–70. 10.1016/j.jneumeth.2008.03.009 18462803

[24] Wang M Wang B Zou J Chen L Shima F Nakamura M A new quantitative evaluation method of Parkinson’s disease based on free spiral drawing. 2010 3rd International Conference on Biomedical Engineering and Informatics (BMEI) Yantai: IEEE (2010). p. 694–8.

[25] Isenkul M Sakar B Kursun O Improved spiral test using digitized graphics tablet for monitoring Parkinson’s disease. Proc. of the Int’l Conf. on e-Health and Telemedicine Istanbul (2014). p. 171–5.

[26] Bewick V Cheek L Ball J. Statistics review 10: further nonparametric methods. Crit Care (2004) 8(3):1. 10.1186/cc2398 15153238 PMC468904

[27] Hallett M Khoshbin S. A physiological mechanism of bradykinesia. Brain (1980) 103(2):301–14. 10.1093/brain/103.2.301 7397480

[28] Raudmann M Taba P Medijainen K Handwriting speed and size in individuals with Parkinson’s disease compared to healthy controls: the possible effect of cueing. Acta Kinesiologiae Universitatis Tartuensis (2014) 20(0):40 10.12697/akut.2014.20.04

[29] Schomaker LRB Plamondon R The relation between pen force and pen-point kinematics in handwriting. Biol Cybern (1990) 63(4):277–89. 10.1007/BF00203451

[30] Majsak MJ Kaminski T Gentile AM Flanagan JR. The reaching movements of patients with Parkinson’s disease under self-determined maximal speed and visually cued conditions. Brain (1998) 121(4):755–66. 10.1093/brain/121.4.755 9577399

